# Supplementary material for: A high-resolution mRNA expression time course of embryonic development in zebrafish
Source: eLife. 2017 Nov 16;6:e30860. doi: 10.7554/eLife.30860 (PMC5690287; doi:10.7554/eLife.30860)
Supplement: Supplementary file 6. [file elife-30860-supp6.zip › biolayout-clusters-files/Cluster097-genes.html]

Cluster097


# Cluster097: Genes

| Ensembl ID | Gene Name | Chr | Start | End | Biotype |
| --- | --- | --- | --- | --- | --- |
| ENSDARG00000036558 | col18a1 | 9 | 42019826 | 42177718 | protein\_coding |
| ENSDARG00000015559 | fstl1a | 1 | 46768491 | 46794128 | protein\_coding |
| ENSDARG00000037361 | kdelr2b | 12 | 947209 | 954562 | protein\_coding |
| ENSDARG00000008867 | rap1b | 4 | 18863845 | 18950362 | protein\_coding |
| ENSDARG00000009724 | rpn2 | 11 | 15090672 | 15162571 | protein\_coding |
| ENSDARG00000075954 | serpinh1a | 10 | 38568512 | 38577952 | protein\_coding |
| ENSDARG00000039041 | sfrp5 | 13 | 39785661 | 39821445 | protein\_coding |
| ENSDARG00000102850 | si:ch211-132i7.2 | 7 | 67523900 | 67524663 | lincRNA |
| ENSDARG00000092881 | si:ch211-223c9.2 | 22 | 29742705 | 29886831 | processed\_transcript |
| ENSDARG00000076144 | si:ch211-74m13.3 | 3 | 54489467 | 54519018 | protein\_coding |
| ENSDARG00000033170 | sult2st1 | 12 | 624354 | 640031 | protein\_coding |
| ENSDARG00000023963 | tpm4a | 22 | 15552293 | 15576521 | protein\_coding |
| ENSDARG00000005216 | zgc:158328 | 15 | 35395278 | 35486665 | protein\_coding |
